# Weighting Low-Intensity MS/MS Ions and *m*/*z* Frequency for Spectral Library Annotation

**DOI:** 10.1021/jasms.3c00353

**Published:** 2024-01-25

**Authors:** Chloe Engler Hart, Tobias Kind, Pieter C. Dorrestein, David Healey, Daniel Domingo-Fernández

**Affiliations:** # Enveda Biosciences, 5700 Flatiron Parkway, Boulder, Colorado 80301, United States; ‡ Collaborative Mass Spectrometry Innovation Center, Skaggs School of Pharmacy and Pharmaceutical Sciences, University of California San Diego, La Jolla, California 92093, United States

## Abstract

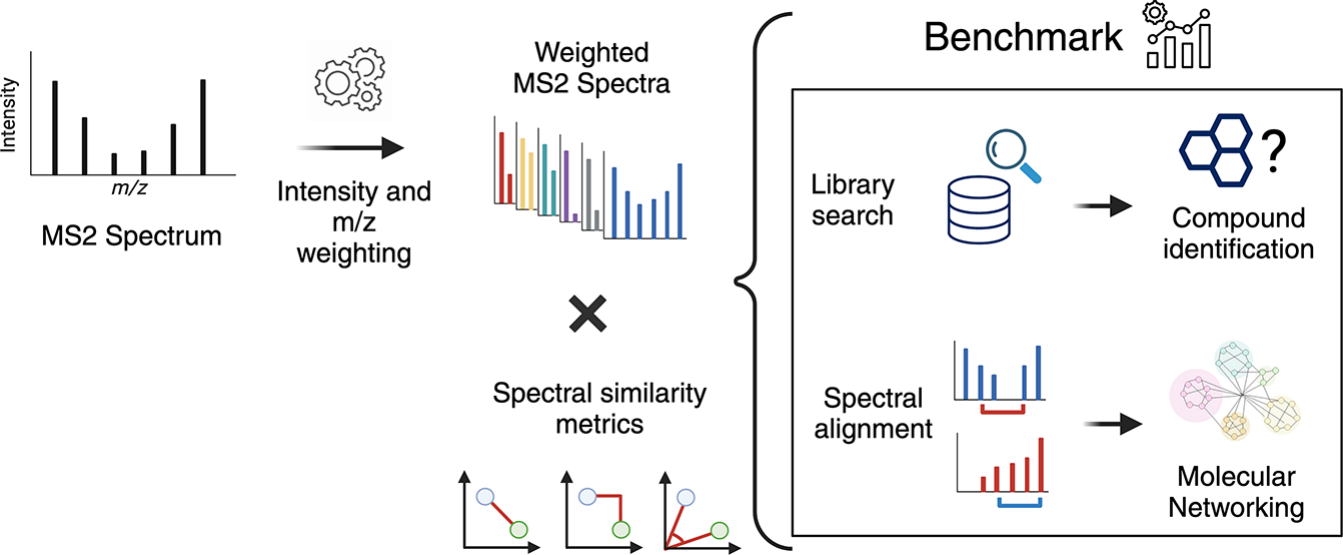

Calculating spectral similarity is a fundamental step in MS/MS data analysis in untargeted metabolomics experiments, as it facilitates the identification of related spectra and the annotation of compounds. To improve matching accuracy when querying an experimental mass spectrum against a spectral library, previous approaches have proposed increasing peak intensities for high *m*/*z* ranges. These high *m*/*z* values tend to be smaller in magnitude, yet they offer more crucial information for identifying the chemical structure. Here, we evaluate the impact of using these weights for identifying structurally related compounds and mass spectral library searches. Additionally, we propose a weighting approach that (i) takes into account the frequency of the *m*/*z* values within a spectral library in order to assign higher importance to the most common peaks and (ii) increases the intensity of lower peaks, similar to previous approaches. To demonstrate our approach, we applied weighting preprocessing to modified cosine, entropy, and fidelity distance metrics and benchmarked it against previously reported weights. Our results demonstrate how weighting-based preprocessing can assist in annotating the structure of unknown spectra as well as identifying structurally similar compounds. Finally, we examined scenarios in which the utilization of weights resulted in diminished performance, pinpointing spectral features where the application of weights might be detrimental.

## 1 Introduction

Mass spectrometry plays a crucial role in the identification and characterization of molecules that are found in biological samples. Tandem mass spectrometry (MS/MS) has emerged as a powerful technique for elucidating the structure of molecules by generating fragmentation patterns and spectra.^1^ To predict the chemical structure, we matched unknown spectra to a library of known spectra using spectral similarity metrics. However, spectral libraries exclusively cover a fraction of the chemical space. For instance, the largest spectral libraries cover less than a million unique structures and the chemical space is estimated to be 10^60^ structures.^2^ Consequently, as spectral libraries expand, optimizing spectral similarity metrics remains a fundamental approach to enhancing the accuracy of annotating unknown spectra.

Although newly introduced machine learning approaches like MS2DeepScore^3^ have been developed, Cosine (dot product) similarity and other alternative metrics such as spectral entropy^4^ are regarded as key measures for evaluating the similarity between pairs of MS/MS spectra. These spectral similarity metrics function by comparing and analyzing fragment ion intensities and peak distributions. One notable advantage of these metrics is their transparency. This transparency enables scientists to explore the fragmentation patterns that contribute to a high similarity score.

Several recent studies have compared the relative performances of different similarity metrics and proposed new ones. In a recent study by Li et al.,^4^ which evaluated the performance of 42 similarity metrics for mass spectral library searches (matching an unknown spectrum against a reference database) across two of the largest libraries (i.e., the National Institute of Standards and Technology (NIST) and MassBank of North America), spectral entropy outperformed the established methods, including cosine similarity. More recently, Bittremieux and colleagues^5^ benchmarked modified cosine similarity, cosine similarity, and neutral loss^6^ on the task of spectral alignment. Unlike library search, which aims to match two near-identical spectra, spectral alignment aims to identify similar spectra corresponding to structurally related compounds that are not necessarily exact matches. Spectral alignment tasks typically operate with larger *m*/*z* tolerance windows than library search tasks and are the methods typically used in molecular networking.^7^

Some of these similarity metrics place a disproportionate emphasis on the highest peaks in spectral similarity calculations. For example, the function for cosine similarity, one of the most commonly used metrics, involves summing products. In this context, each summand is the product of two intensities. When two intensities in a product are high, their contribution to the total sum is substantial. Conversely, when the two intensities in a product are low, even if they align well, their contribution to the sum is much lower. To address this concern, Sokolow et al.^8^ were, to our knowledge, the first researchers to introduce scaling the intensities; in this case, they proposed applying the square root of an intensity multiplied by its *m*/*z* value. This adjustment aimed to enhance the results of mass spectral library searches by boosting peak intensities for low peaks, particularly focusing on the high *m*/*z* ranges. Building upon this work, Stein and Scott^9^ conducted a benchmarking exercise using the 1992 release of the NIST database. They discovered that cosine similarity, with intensities raised to the power of 0.6 times their *m*/*z* value cubed, emerged as the most effective metric for conducting library searches. Similarly, Stein^10^ explored the idea of increasing the weights for low *m*/*z* values.

In the realm of gas chromatography–mass spectrometry (GC–MS) and in a more contemporary context, Horai et al.^11^ reported that the optimal weight factor is a function of the square root of the intensity and the square of its *m*/*z* value. Kim et al.^12^ proposed a method for determining optimal weights for compound identification also in the context of GC–MS. They identified 0.53 as the optimal weight for intensity scaling and 1.3 for mass weighting, utilizing the NIST database and aligning with prior findings. More recently, Li and Fiehn^13^ expanded upon their novel concept of spectral entropy similarity^4^ by incorporating weighted intensities based on spectral entropy. Their method keeps the intensity values unaltered in spectra with a spectral entropy of at least three. If the spectral entropy is below 3, the intensity is raised to the power of 0.25(1 + *S*) (see eq 1), where *S* is the spectral entropy of the spectrum. Lastly, weighting based on the peak occurrence has also been proposed in the field of proteomics during spectral clustering.^14^ In this particular case, infrequent peaks are upweighted rather than frequent peaks, conceptually attempting to emphasize more discriminative signals.
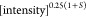
1

In this study, we investigate the impact of applying weights to spectral alignment as a preprocessing for the purpose of identifying structurally related compounds and mass spectral library searches. We build upon a variety of similarity metrics and data sets utilized in recent benchmarks.^4,5^ We also introduce a weighting method for spectral intensity values that considers the relative frequency of *m*/*z* values in a reference database. This approach involves weighting up peaks with common *m*/*z* values. Alongside these weights, we assign higher weights to peaks with low intensities, similar to the methods mentioned earlier. Regarding the task of identifying structurally related compounds, we demonstrate enhancements across 42 distinct spectral similarity metrics through the application of our weighting strategy. Finally, we demonstrate that applying weights also improves results when querying spectral libraries.

## 2 Methods

### 2.1 Weighting Intensities and *m*/*z* Frequency in MS/MS Spectra

In this subsection, we propose two types of weighting preprocessing methods using spectra intensities and *m*/*z* frequencies that we describe below.

#### 2.1.1 Weighting Intensities

Normalizing the intensities is a common preprocessing step. Previous studies,^8−10^ used intensity weighting functions following the format of eq 2 which considers peak intensities and *m*/*z* values (both are raised to the power of *n* and *m*, respectively). We adjusted this function by normalizing peak intensity and incorporating the frequencies of *m*/*z* values (eq 3), where g is a normalization function and f(*m/z*) represents the frequency of *m/z* values (graphed in Supplementary Figure 2). The specifics of each component of this function are elaborated on in subsections 2.1.2 and 2.1.3.
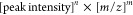
2
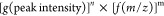
3

#### 2.1.2 Rescaling Low Intensity Peaks

Drawing on previous research,^8−10^ we implemented weights designed to amplify peaks with lower intensities within a given spectrum. By definition, many similarity metrics, such as cosine similarity, put disproportionate emphasis on peaks with higher intensities. These intensity-based weighting methods were introduced to mitigate the dominance of these high-intensity peaks in the calculated similarity scores. To address this issue, Sokolow^8^ first introduced an intensity weighting function that raised the intensity values to a power of *n* (as shown on the left side of eq 2). Various values for n have been utilized in the literature including *n* = 0.5 (Sokolow et al.;^8^ Horai et al.^11^), *n* = 0.6 (Stein and Scott^9^), and *n* = 0.53 (Kim et al.^12^). We adopt a similar approach for intensity weighting using *n* = 0.25. However, we add an additional normalization step by dividing the intensities of each spectrum by the highest intensity value. This normalization enabled us to assign weights to peaks based on their relative intensity in comparison to the peak with the highest intensity rather than relying solely on their raw intensity values (as illustrated in Supplementary Figure 1A,B).

#### 2.1.3 Rescaling Based on *m*/*z* Peak Occurrence

In addition to weighting low intensity peaks, we hypothesized that weighting intensities approximating the density of peaks across the *m*/*z* range could enhance the performance of similarity metrics. We implemented this by calculating the frequency of occurrence for the *m*/*z* values in a reference library. The *m*/*z* range had previously been employed for intensity weighting.^8−10^ However, prior weighting approaches had multiplied the intensity weights (described in section 2.1.2) by the corresponding *m*/*z* value raised to some power *m* (as shown on the right side of eq 2). In our methodology, we take a different approach by multiplying our intensity weights by the relative frequency of the corresponding *m*/*z* value raised to the power of 0.25 (as shown on the right side of eq 3). This weighting approach accentuates intensities linked to frequently occurring *m*/*z* values (see an example in Supplementary Figure 1). To determine the relative *m*/*z* frequency values, we employed the GNPS^15^ data set from Bittremieux et al.^5^ We identified the number of spectra within the data set exhibiting a peak at individual *m*/*z* values (rounded to 1 decimal place) (Supplementary Figure 2). Subsequently, we calculated the relative frequency for each *m*/*z* value by considering the percentage of spectra in the data set exhibiting a particular *m*/*z* value.

### 2.2 Spectrum Similarity Metrics

We benchmark the best-performing similarity metrics identified by Li et al.^4^ in their benchmark, including spectral entropy, cosine similarity, and Bhattacharya 1 similarity (Supplementary Table 1).

### 2.3 Applications

#### 2.3.1 Spectral Alignment for Identifying Structurally Related Compounds

To evaluate the spectrum similarity measures on how well they align structurally related compounds, we leveraged the benchmark data set released by Bittremieux et al.^5^ This data set consists of 495,600 MS/MS spectra from small molecules available in GNPS.^15^ We applied filtering steps similar to those in the previous application conducted in the original benchmark (Supplementary Table 2). Given the large number of spectrum pairs resulting from all possible combinations of the data set, Bittremieux et al.^5^ benchmarked the similarity metrics on 10 million randomly selected MS/MS spectrum pairs. Thus, we benchmarked all similarity metrics described in subsection 2.2 on the same 10 million spectrum pairs with and without applying our weighting approach. Lastly, following the original evaluation, we compared the resulting similarity scores on these 10 million pairs to the chemical similarity between the two molecules, which is defined as the Tanimoto coefficient of the RDKit topological fingerprints (using default settings on v2023.03.1).^16^

#### 2.3.2 Mass Spectral Library Search Using GNPS

**Figure 1:**
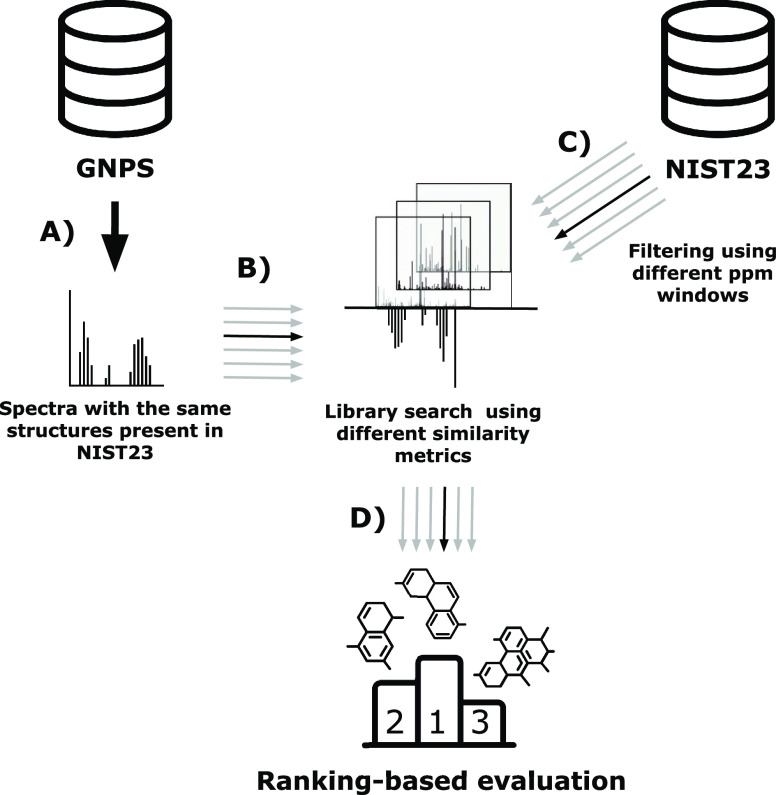
Design of the mass spectral library search task. (A) The small molecule data set from GNPS was used as the query, following the same filtering steps previously utilized for the preceding spectral alignment task. The query data set was subsequently filtered to contain structures present in NIST, which are considered positive hits in the evaluation. (B) Mass spectral library search using a variety of similar metrics against NIST. (C) Spectral matching is conducted using different ppm windows. (D) Evaluation of the top K matches.

To evaluate our weighting approach on a retrieval task (mass spectral library search), we employed NIST23, a high-quality database of reference spectra, also used by the previous benchmark by Li et al.^4^ in its prior version. We also leveraged an independent data set, GNPS, which was described in the previous subsection. We filtered both data sets using a similar filtering procedure as that of Bittremieux et al.^5^ (Supplementary Table 2). We filtered the GNPS data set to only include spectra for molecules that were present in the NIST23 data set (i.e., the ones which we designate as true positives) using the first 14 characters of their InChIKeys (Figure 1A). In total, we found 25,437 spectra in the filtered GNPS data from 2,776 unique molecules that are also present in NIST23. Next, for each of these 25,437 spectra, we queried against NIST23 (Figure 1B) using a precursor *m*/*z* window of 10 ppm with modified cosine similarity, in line with Li et al.^4^ We also queried NIST23 using precursor windows of 100 and 5,000 ppm to demonstrate how each method would scale when more diverse spectra were included in the queries (Figure 1C).

Similar to Stein and Scott,^9^ we evaluated the performance of each spectral similarity metric for library search using a rank-based metric (i.e., precision@K) (Figure 1D). We chose precision@K to emulate the process that is typically used in library searches for unidentified spectra. In such searches, one exclusively focuses on the top-ranked results, as manually examining further results becomes impractical due to time constraints. In other words, our evaluation approach assesses similarity metrics based on their ability to return the most accurate matches within the highest ranked spectra. This prioritizes similarity metrics that are better suited for real-world library searches. Additionally, we report AUC-ROC scores, in line with Li et al.^4^

### 2.4 Implementation

Scripts and notebooks are written in Python using open source libraries such as matchms,^17^ RDKit,^16^ NumPy,^18^ Pandas,^19^ and SciPy.^20^ To calculate spectral similarities, we leveraged the previous implementations released in the benchmarks by Li et al.^4^ and Bittremieux et al.^5^ Data, scripts and notebooks are available at https://github.com/enveda/weighting-spectral-similarity.

## 3 Results

### 3.1 Spectral Alignment for Identifying Structurally Related Compounds

Spectral alignment is crucial for identifying groups of similar compounds used in applications, such as molecular networking. This involves calculating the similarity between pairs of spectra, followed by the representation of compounds from one or more samples in a network. Each node in the network corresponds to a compound with edges depicting their spectral similarity. This network-based approach allows researchers to identify related compounds, infer potential biosynthetic pathways, and highlight differences or similarities in metabolic profiles between samples.

**Figure 2:**
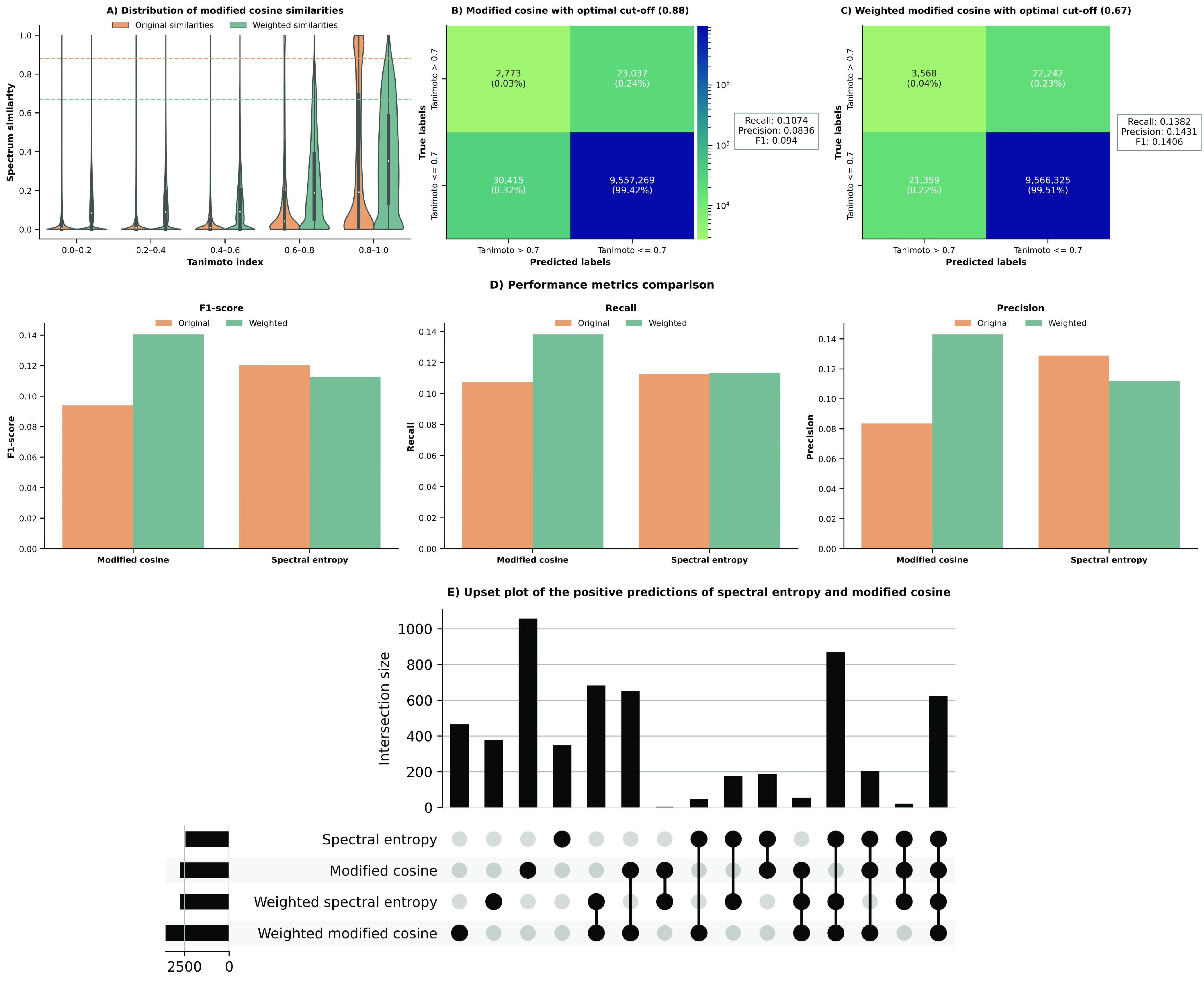
(A) Distribution of the spectral similarity for unweighted and weighted modified cosine similarity. The plot represents the similarity scores across 10 million pairs of spectra binned in relation to the structural similarities of the pair of molecules, measured using the Tanimoto coefficient. Higher Tanimoto coefficients (0.6–1) correspond to pairs of compounds with high structural similarity, and vice versa. The two horizontal lines represent the optimal cut-offs for predicting if a pair of spectra is structurally similar (label 1/Tanimoto coefficient > 0.7) or not (label 0/Tanimoto coefficient ≥ 0.7) based on the F1-score for each variant (i.e., 0.88 for unweighted and 0.67 for weighted). The rest of the similarity metrics are found in Supplementary Figure 3B,C) Contingency tables for modified cosine unweighted (B) and weighted (C) using the previously mentioned optimal cut-offs based on the F1-score. (D) Comparison of the performance metrics for modified cosine and spectral entropy. The optimal cut-offs for spectral entropy unweighted and weighted were 0.55 and 0.56, respectively. The performances for the remaining metrics are shown in Supplementary Table 4. (E) Upset plot showing the overlap of the true positives yielded by spectral entropy and modified cosine..

In this first application, we evaluated whether applying the proposed weights to the most commonly used similarity metrics yielded enhanced outcomes in the identification of structurally related compounds. To do so, we replicated the recent benchmark presented by Bittremieux et al.^5^ and expanded it with over 40 additional similarity metrics. Our results indicate that applying weights on the intensities and *m*/*z* frequencies improves spectral alignment. This improvement is evident as the spectral similarities more closely resemble their corresponding Tanimoto coefficients after applying weights (Figure 2 and Supplementary Figure 3). For example, when weights are applied to modified cosine similarity, the top-performing metric reported by Bittremieux et al.,^5^ we observe a gradual rise in spectral similarity scores as the Tanimoto coefficient increases from 0 to 1 (Figure 2A). This trend is observed across most of the benchmarked metrics, including spectral entropy, Bhattacharya 1, and fidelity similarity, metrics which showcased superior performance in the spectral library search benchmark by Li et al.^4^ (Supplementary Figure 3).

Focusing on the spectral similarities for closely related structures, we noted a substantial increase in the spectral similarity scores when weights were applied. To confirm this, we calculated the optimal spectral similarity threshold to achieve the maximum F1 score in the case of modified cosine similarity for both distributions without and with weights. This effectively enabled us to quantify how accurately can a spectral similarity cutoff separate between structurally similar and dissimilar pairs, which we define using a standard threshold of 0.7 Tanimoto coefficient.^21^ In the case of the original spectral similarities obtained with modified cosine, we found that the optimal threshold was 0.88, which achieved an F1-score of 0.094. Similarly, commonly used cut-offs such as 0.7 yielded a F1 score of 0.0854. Using weights on modified cosine with the optimal threshold (0.67) improved the F1-score significantly to 0.14. To contextualize these results in the classical application of spectral alignment, molecular networking, using these optimal cut-offs, applying weights yields approximately 30% accurately predicted edges (i.e., 3,568 vs 2,773) while reducing the number of false positives by approximately 800 edges (Figure 2B,C). Similarly, we also observed the advantage of applying weights when increasing the Tanimoto coefficient threshold (e.g., a Tanimoto threshold of 0.8 yielded a F1-score of 0.089 with the original similarities (optimal cutoff = 0.95) and 0.125 using weights (optimal cutoff = 0.74)) and optimizing the cutoff based on F2-score in order to favor recall over precision. Lastly, the advantage of using weights is also highlighted for pairs of spectra with lower structural similarity, which constitutes the majority of cases. Importantly, both applying and not applying weights to pairs of structures with low structural similarity (i.e., Tanimoto coefficient <0.2) do not yield high spectral similarities (i.e., false positives). However, for pairs with a Tanimoto coefficient between 0.4 and 0.8, not applying weights still yields low spectral similarities, while applying weights significantly shifts the distributions upward for these pairs with a “medium” structural similarity.

Additionally, we evaluated the overlap of the true positives for the weighted and unweighted versions of spectral entropy and modified cosine (Figure 2E). The method with the highest number of accurate predictions is modified cosined (weighted), followed by spectral entropy (weighted). We would like to note that it is not surprising that, across all methods, modified cosine has the highest number of accurate predictions, since it is the only method we have benchmarked that finds the best possible matches by adjusting spectral alignment. Nevertheless, it is noteworthy that spectral entropy (weighted) yielded a higher number of true positives than modified cosine (unweighted). Furthermore, all four methods exhibited a large overlap (Figure 2E, last column). Lastly, when examining specific intersections, we found that the largest overlap between two distinct methods was between modified cosine (weighted) and spectral entropy (weighted), closely followed by modified cosine (weighted) and modified cosine (unweighted).

**Figure 3:**
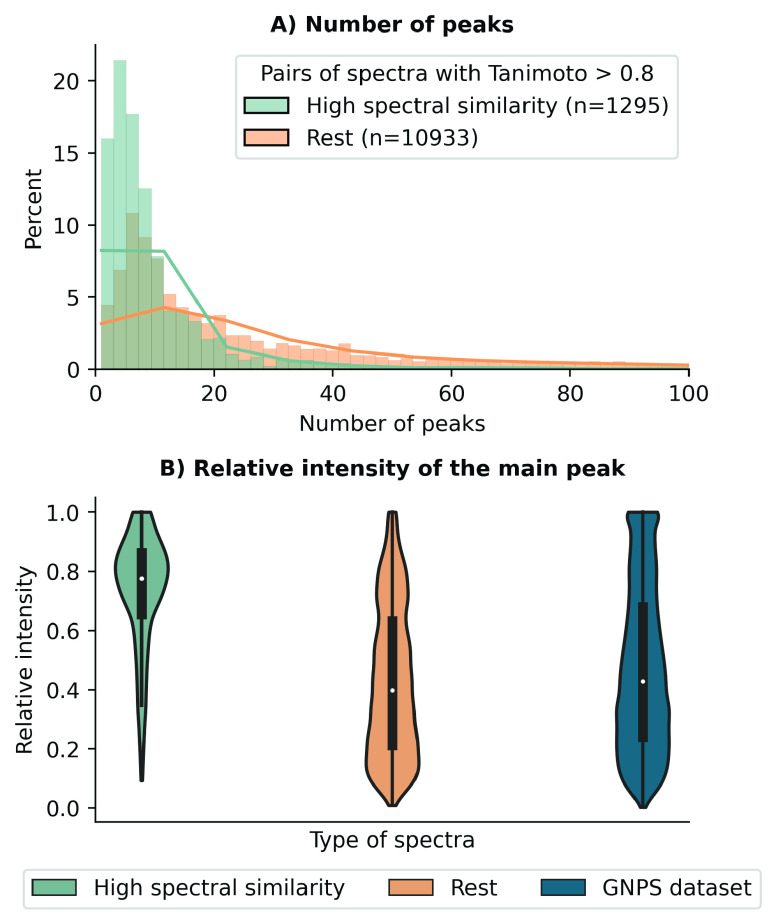
(A) Distribution of the number of peaks per spectrum within the pairs with a high Tanimoto coefficient (0.8–1) grouped by pairs with a cosine similarity higher than 0.95 (green) and the rest (orange). The plot shows that pairs of spectra corresponding to structurally similar compounds (Tanimoto coefficient 0.8–1) that exhibit a high spectral similarity (>0.95) generally have a lower number of peaks compared with the rest of structurally similar compounds (Tanimoto coefficient 0.8–1). (B) Distribution of the relative intensity of the main peak. The violin plots show the distribution of the relative intensities for three groups: (left) pairs of spectra with >0.95 spectral similarity using modified cosine similarity within the bin of Tanimoto coefficient 0.8–1, (center) rest of pairs of spectra with less than 0.95 spectral similarity using modified cosine similarity within the bin of Tanimoto coefficient 0.8–1, and (right) distribution of all the spectra in the GNPS data set.

Given that applying weights decreases the number of pairs with a very high spectral similarity (Figure 2A) (e.g., spectral similarity >0.95 (1,295 original vs 89 with weights)), it is not surprising that the optimal cutoff when applying weights is approximately 0.2 lower compared to the original modified cosine similarities. While this only occurs in the case of modified cosine, it reveals the importance of lowering the typically applied cut-offs, when using weights. To investigate why the number of pairs with a high spectral similarity is so low when applying weights, we explored the characteristics of this subset of spectra and found that pairs with high similarity using modified cosine similarity typically have (i) a low number of peaks compared to the rest of the spectra (Figure 3A), a main peak representing more than 0.7 of the relative total intensity (Figure 3B).

These findings are to be expected when taking into account how the weights operate since in these pairs of spectra we find a single dominant peak with high intensity, which is aligned by modified cosine. When applying the weights, the contribution of the relative intensity of this main peak to the overall score diminishes, as the nonmatching smaller peaks are now higher. Thus, the cosine similarity decreases when weights are applied, since previously the main peaks in each spectrum were perfectly aligned and the handful of low peaks did not contribute to the cosine similarity score. When examining the majority of these pairs, we found an over-representation of glycosidic flavonoids and other compounds with glycosidic bonds (Supplementary Figure 4). Such compounds typically present the previously mentioned characteristics, as the glycosidic bond is easily broken but the rest of the flavonoid scaffold cannot be broken. In conclusion, these insights can serve as a criterion to know when to apply weights or not. For instance, weights are generally not required if modified cosine similarity yields a match with a score close to 1, or when aligning spectra with only a few peaks or a single dominant peak.

Additionally, we investigated the impact of different weight functions on the intensities. First, we explored the effect of setting all the intensities to 1 (Supplementary Figure 5). Not surprisingly, for pairs with high Tanimoto scores, binarizing the intensities to 0 or 1 depending on the appearance of a peak did not yield better results than applying most other reported weights but still achieved a higher mean compared with the unweighted spectral similarities (Supplementary Figure 6). Next, we explored using different weights, including the ones proposed by Sokolow et al.^8^ (intensity weight = 0.5, *m*/*z* weight = 0.5 in eq 2), Stein and Scott^9^ (intensity weight = 0.6, *m*/*z* weight = 3 in eq 2), Horai et al.^11^ (intensity weight = 0.5, *m*/*z* weight = 2 in eq 2), Kim et al.^12^ (intensity weight = 0.53, *m*/*z* weight = 1.3 in eq 2) and other commonly used weights (intensity weight = 0.5, *m*/*z* weight = 0). Similar to our proposed weights, all these previously mentioned weights yield similarity scores that are closer to their respective Tanimoto coefficient bins, compared to the original similarities. We then compared these previously reported weights to ours using Kullback–Leibler (KL) divergence, a statistical test that allowed us to compare how close the distribution of the spectral similarities was to the distribution of the Tanimoto coefficients (Supplementary Table 3, Supplementary Figure 6). Our results demonstrate that our weights best resemble the Tanimoto coefficients.

Lastly, we explored the contribution of the *m*/*z* frequency weights with respect to intensity rescaling. As expected, rescaling based on *m*/*z* peak occurrence exhibited a minimal impact compared with rescaling based on peak intensity, since the *m*/*z* frequency variability is much lower for most spectra (Supplementary Figure 1). Consequently, we tested the impact of the *m*/*z* frequency rescaling and found that *m*/*z* frequency weights do improve results and improve KL divergence (KL divergence = 0.16 only intensity weights, and KL divergence = 0.112 intensity + *m*/*z* weights) (Supplementary Table 3). In conclusion, our study has demonstrated the effectiveness of both proposed weighting strategies, namely, intensity and *m*/*z* frequency, in enhancing the performance of spectra alignment tasks. Among the various weight modifications, our approach most accurately approximated structural similarity.

### 3.2 Mass Spectral Library Search

A reference library in mass spectrometry is a collection of mass spectra from known compounds. It serves as a valuable resource for compound identification as it allows researchers to match experimental spectra against the reference spectra for confident identification of compounds. The similarity metric used for querying in a reference library is key because experimental data may exhibit small shifts in *m*/*z* values and intensities due to various factors such as instrument calibration, ionization efficiency, or sample preparation.^22^ As a result of distinct approaches taken by different similarity metrics to align spectra and correct for these variations, there can be significant differences in the best matches returned by each similarity metric.^4^

**Figure 4:**
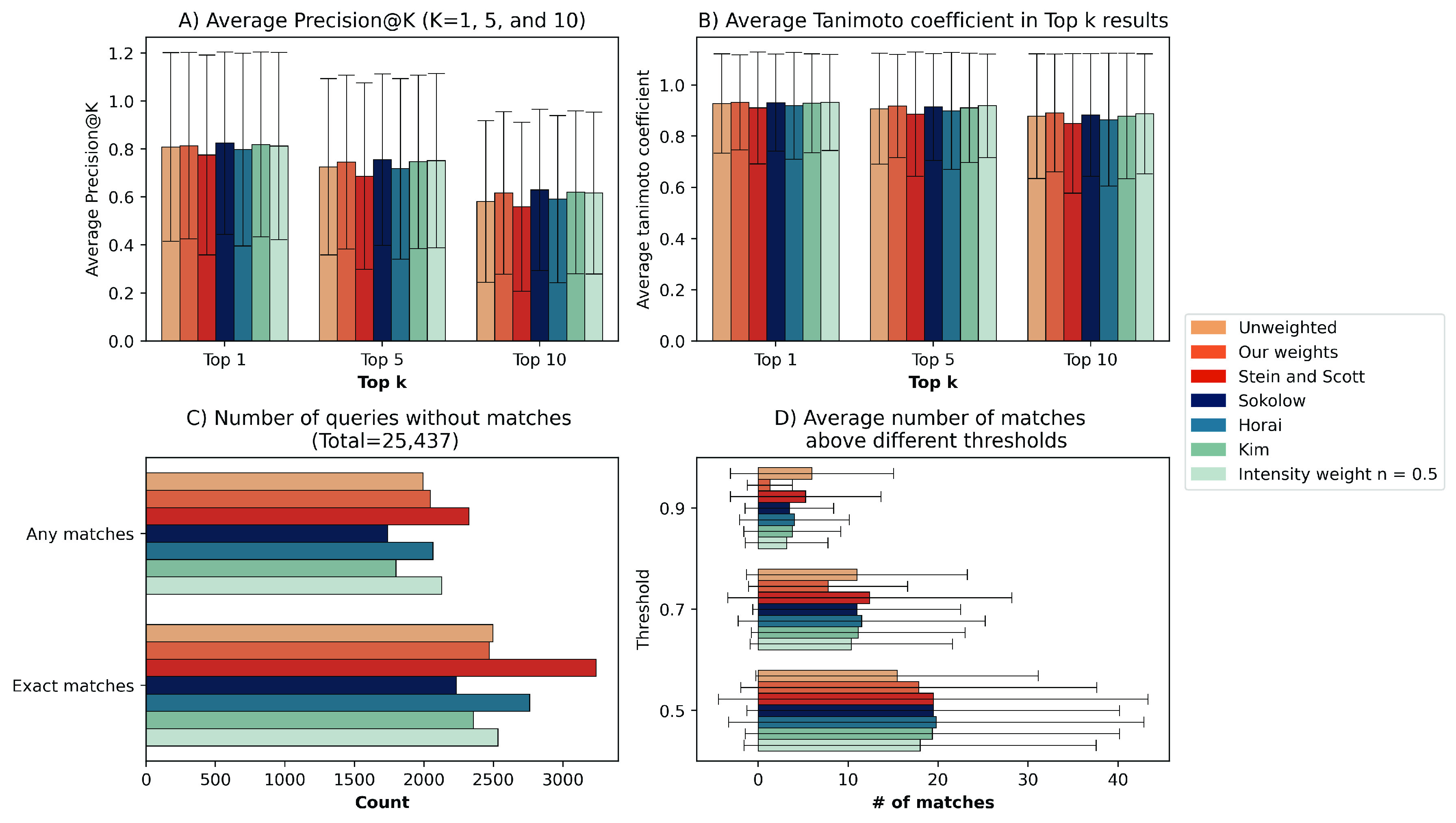
Overview of the results on the mass spectral library search task. (A) Average precision@K values on the top 1, 5, and 10 matches using different proposed weights in the literature and our proposed weights. Library search is run using modified cosine similarity with a parts per million window of 10. Their performances are compared against unweighted scores (baseline). (B) Average Tanimoto coefficients of the structures within the top 1, 5, and 10 matched spectra. (C) Number of queries that did not return any match with a spectral similarity higher than 0.5 out of the total 25,437 queries. The bottom set of bars represents the number of queries without any exact matches. The upper set represents the number of queries without any matches. (D) Average number of matched spectra above different thresholds for spectral similarity (i.e., 0.5, 0.7, 0.9).

In this subsection, we simulated a real-world library search application by leveraging GNPS and NIST spectra generated from the same chemical structures to benchmark different weighting methods using a modified cosine similarity (Figure 2). First, we explored the precision of the top matches in this retrieval task (Figure 4A). This metric quantifies the number of true positives within the top K hits, making it a reliable way to evaluate the quality of returned matches. Here, we found that the weights from Sokolow et al.^8^ outperformed the other weights in this task closely followed by our weights and the weights from Kim et al.^12^ Surprisingly, Stein and Scott^9^ and Horai et al.^11^ performed worse than the unweighted modified cosine similarity (baseline). This contrasts with the results from the spectral alignment task, where both weighted methods outperformed the unweighted method.

Zooming into the top matches, we observed that our proposed weights, the Sokolow et al.^8^ weights, and the intensity weights with *n* = 0.5 yielded the most similar molecules to the one queried, closely followed by the Kim et al.^12^ weights (Figure 4B). Additionally, we also evaluated our results using ROC-AUC, although, as mentioned in the Methods, we believe the ranked-based metric is more informative. When evaluating using ROC-AUC, our weights slightly outperformed the weights from Sokolow et al.^8^ (Supplementary Figure 7). Subsequently, we analyzed the number of queries that failed to yield any match with a spectral similarity higher than 0.5 for each method (Figure 4C). Overall, all methods returned matches for approximately 90% of the 25,437 queries. However, we observed differences between methods. For example, the weights from Sokolow et al.,^8^ Kim et al.^12^ and our weights had fewer queries without an exact match than the unweighted method, while the other methods had more queries without an exact match.

Lastly, we investigated the average number of matches using different thresholds (i.e., 0.5, 0.7, 0.9). Similar to the spectral alignment results, we found that the original cosine similarity (i.e., unweighted baseline) returned the largest number of matches above 0.9 similarity (Figure 4D). However, the number of matches compared to the weighted methods decreases as the threshold gets lower. Furthermore, we evaluated how the results changed when using different ppm windows. We performed similar queries with ppm windows of 100 ppm and 5,000 ppm to access the scalability of the weighting methods for more varied molecule sets (Supplementary Figure 8). Unsurprisingly, the overall precision for all weighting methods decreased as we expanded the searches to more molecules with larger ppm windows. However, the precision scores for queries employing our weights experienced a comparatively slower decrease compared to those for the other weighting methods. These results are consistent with the results from the spectral alignment task where there was a bigger variety of molecules since no parts per million window was applied. In that task the weighted methods were the top-performing approaches.

## 4 Conclusion

Here, we demonstrated that weighting the intensities can assist in the identification of analogs and can improve the annotation of unknown compounds, regardless of the similarity metric employed. Furthermore, we introduced a weighting method for spectral intensity values that accounts for the relative frequency of *m*/*z* values, which can be used in conjunction with other weights. In the task of identifying structurally related compounds, we demonstrated improvement for the most widely used spectral similarity metrics such as modified cosine similarity by applying our proposed weights, improving upon previous weighting methods. Similarly, on the library search task, we demonstrated that applying weights leads to improved performance and specifically, our weights are on par with the other best-performing weights proposed by Sokolow et al.^8^ Finally, we have explored examples where the application of weights has resulted in diminished performance, identifying spectral characteristics where the implementation of weights may not yield advantages.

This work is not without its limitations. First, while applying weights for spectral alignment demonstrates a predominantly positive effect on the results across all similarity metrics, it tends to perform worse when using modified cosine similarity yields a high spectral similarity (>0.95). However, we identified spectral characteristics in these cases (i.e., spectra with few peaks, spectra with a single major peak, and presence of a glycosidic bond) where applying the weights typically decreases spectral similarity for pairs of structurally similar compounds. Thus, to determine whether weights should be applied, we recommend investigating these factors in the query spectra first. Second, the spectral alignment task is based on the original 10 million pairs of spectra used by Bittremieux et al.,^5^ which are derived from all the combinations of spectra present in GNPS. Ideally, this task should be conducted on all combinations, but computational time constraints make it infeasible. We verified that the subset is a representative sample of the full GNPS data set. Third, in the case of the library search task, we presented a real application where nonoverlapping spectra from GNPS were used to query the NIST database. However, after the filtering steps, we were only able to benchmark over 25,000 queries belonging to 2,776 unique molecules. We hypothesize that this limited data set size coupled with the observation that many queries either yielded no matches or quickly reached a saturation point in the number of hits are the primary reasons we exclusively observed limited improvement using weights for the spectral alignment task. Finally, we note that applying weights inherently makes the spectral similarity calculations more abstract and hampers their interpretability.

We aim to pursue several directions for our work in the future. First, there might exist a variety of optimal weights based on the type of molecule that we are querying/aligning. Thus, future endeavors could investigate the optimal weights for different compound classes. However, unveiling this array of optimal weights is perhaps a task more suited for deep learning approaches, such as MS2DeepScore.^3^ Second, we have expanded our benchmarks beyond NIST by incorporating GNPS spectral libraries; nevertheless, as new resources emerge and both of these databases undergo updates, our proposed weights and the previous ones must be reassessed. Third, we believe that harnessing collision energy information could potentially enhance the outcomes of the spectral alignment task. Since weights could potentially be adjusted based on the employed energy, this information could potentially offer improvements. Unfortunately, we were unable to utilize this information since it is generally not available within the GNPS data set.

## Supporting Information Available

Supplementary Figures 1–8 comparing weighting functions and showing spectral similarities; Supplementary Tables 1–4 (PDF)

## References

[ref1] Alseekh S.; Aharoni A.; Brotman Y.; Contrepois K.; D’Auria J.; Ewald J.; C Ewald J.; Fraser P. D.; Giavalisco P.; Hall R. D.; Heinemann M.; Link H.; Luo J.; Neumann S.; Nielsen J.; Perez de Souza L.; Saito K.; Sauer U.; Schroeder F. C.; Schuster S.; Siuzdak G.; Skirycz A.; Sumner L. W.; Snyder M. P.; Tang H.; Tohge T.; Wang Y.; Wen W.; Wu S.; Xu G.; Zamboni N.; Fernie A. R. Mass spectrometry-based metabolomics: a guide for annotation, quantification and best reporting practices. Nat. Methods 2021, 18 (7), 747–756. 10.1038/s41592-021-01197-1. 34239102 PMC8592384

[ref2] Reymond J. L. The chemical space project. Acc. Chem. Res. 2015, 48 (3), 722–730. 10.1021/ar500432k. 25687211

[ref3] Huber F.; van der Burg S.; van der Hooft J. J. J.; Ridder L. MS2DeepScore: a novel deep learning similarity measure to compare tandem mass spectra. Journal of cheminformatics 2021, 13 (1), 84 10.1186/s13321-021-00558-4. 34715914 PMC8556919

[ref4] Li Y.; Kind T.; Folz J.; Vaniya A.; Mehta S. S.; Fiehn O. Spectral entropy outperforms MS/MS dot product similarity for small-molecule compound identification. Nat. Methods 2021, 18 (12), 1524–1531. 10.1038/s41592-021-01331-z. 34857935 PMC11492813

[ref5] Bittremieux W.; Schmid R.; Huber F.; van der Hooft J. J. J.; Wang M.; Dorrestein P. C. Comparison of cosine, modified cosine, and neutral loss based spectrum alignment for discovery of structurally related molecules. J. Am. Soc. Mass Spectrom. 2022, 33 (9), 1733–1744. 10.1021/jasms.2c00153. 35960544

[ref6] Aisporna A.; Benton H. P.; Chen A.; Derks R. J. E.; Galano J. M.; Giera M.; Siuzdak G. Neutral loss mass spectral data enhances molecular similarity analysis in METLIN. J. Am. Soc. Mass Spectrom. 2022, 33 (3), 530–534. 10.1021/jasms.1c00343. 35174708 PMC10131246

[ref7] Watrous J.; Roach P.; Alexandrov T.; Heath B. S.; Yang J. Y.; Kersten R. D.; van der Voort M.; Pogliano K.; Gross H.; Raaijmakers J. M.; Moore B. S.; Laskin J.; Bandeira N.; Dorrestein P. C. Mass spectral molecular networking of living microbial colonies. Proc. Natl. Acad. Sci. U. S. A. 2012, 109 (26), E1743-E1752 10.1073/pnas.1203689109. 22586093 PMC3387089

[ref8] Sokolow S., Karnofsky J., Gustafson P. (Finnigan Corp. San Jose, CA, USA 1978) The Finnigan library search program: Finnigan application report; Finnigan Corp., San Jose, CA, USA.

[ref9] Stein S. E.; Scott D. R. Optimization and testing of mass spectral library search algorithms for compound identification. J. Am. Soc. Mass Spectrom. 1994, 5 (9), 859–866. 10.1016/1044-0305(94)87009-8. 24222034

[ref10] Stein S. E. Chemical substructure identification by mass spectral library searching. J. Am. Soc. Mass Spectrom. 1995, 6 (8), 644–655. 10.1016/S1044-0305(05)80054-6. 24214391

[ref11] Horai H.; Arita M.; Kanaya S.; Nihei Y.; Ikeda T.; Suwa K.; Ojima Y.; Tanaka K.; Tanaka S.; Aoshima K.; Oda Y.; Kakazu Y.; Kusano M.; Tohge T.; Matsuda F.; Sawada Y.; Hirai M. Y.; Nakanishi H.; Ikeda K.; Akimoto N.; Maoka T.; Takahashi H.; Ara T.; Sakurai N.; Suzuki H.; Shibata D.; Neumann S.; Iida T.; Tanaka K.; Funatsu K.; Matsuura F.; Soga T.; Taguchi R.; Saito K.; Nishioka T. MassBank: a public repository for sharing mass spectral data for life sciences. Journal of mass spectrometry 2010, 45 (7), 703–714. 10.1002/jms.1777. 20623627

[ref12] Kim S.; Koo I.; Wei X.; Zhang X. A method of finding optimal weight factors for compound identification in gas chromatography–mass spectrometry. Bioinformatics 2012, 28 (8), 1158–1163. 10.1093/bioinformatics/bts083. 22333245 PMC3324511

[ref13] Li Y.; Fiehn O. Flash entropy search to query all mass spectral libraries in real time. Nature Methods. 2023, 20, 1475 10.1038/s41592-023-02012-9. 37735567 PMC11511675

[ref14] The M.; Käll L. MaRaCluster: A fragment rarity metric for clustering fragment spectra in shotgun proteomics. J. Proteome Res. 2016, 15 (3), 713–720. 10.1021/acs.jproteome.5b00749. 26653874

[ref15] Wang M.; Carver J. J.; Phelan V. V.; Sanchez L. M.; Garg N.; Peng Y.; Nguyen D. D.; Watrous J.; Kapono C. A.; Luzzatto-Knaan T.; Porto C.; Bouslimani A.; Melnik A. V.; Meehan M. J.; Liu W. T.; Crüsemann M.; Boudreau P. D.; Esquenazi E.; Sandoval-Calderón M.; Kersten R. D.; Pace L. A.; Quinn R. A.; Duncan K. R.; Hsu C. C.; Floros D. J.; Gavilan R. G.; Kleigrewe K.; Northen T.; Dutton R. J.; Parrot D.; Carlson E. E.; Aigle B.; Michelsen C. F.; Jelsbak L.; Sohlenkamp C.; Pevzner P.; Edlund A.; McLean J.; Piel J.; Murphy B. T.; Gerwick L.; Liaw C. C.; Yang Y. L.; Humpf H. U.; Maansson M.; Keyzers R. A.; Sims A. C.; Johnson A. R.; Sidebottom A. M.; Sedio B. E.; Klitgaard A.; Larson C. B.; P C. A. B.; Torres-Mendoza D.; Gonzalez D. J.; Silva D. B.; Marques L. M.; Demarque D. P.; Pociute E.; O’Neill E. C.; Briand E.; Helfrich E. J. N.; Granatosky E. A.; Glukhov E.; Ryffel F.; Houson H.; Mohimani H.; Kharbush J. J.; Zeng Y.; Vorholt J. A.; Kurita K. L.; Charusanti P.; McPhail K. L.; Nielsen K. F.; Vuong L.; Elfeki M.; Traxler M. F.; Engene N.; Koyama N.; Vining O. B.; Baric R.; Silva R. R.; Mascuch S. J.; Tomasi S.; Jenkins S.; Macherla V.; Hoffman T.; Agarwal V.; Williams P. G.; Dai J.; Neupane R.; Gurr J.; Rodríguez A. M. C.; Lamsa A.; Zhang C.; Dorrestein K.; Duggan B. M.; Almaliti J.; Allard P. M.; Phapale P.; Nothias L. F.; Alexandrov T.; Litaudon M.; Wolfender J. L.; Kyle J. E.; Metz T. O.; Peryea T.; Nguyen D. T.; VanLeer D.; Shinn P.; Jadhav A.; Müller R.; Waters K. M.; Shi W.; Liu X.; Zhang L.; Knight R.; Jensen P. R.; Palsson B. O.; Pogliano K.; Linington R. G.; Gutiérrez M.; Lopes N. P.; Gerwick W. H.; Moore B. S.; Dorrestein P. C.; Bandeira N. Sharing and community curation of mass spectrometry data with Global Natural Products Social Molecular Networking. Nature biotechnology 2016, 34 (8), 828–837. 10.1038/nbt.3597. PMC5321674 27504778

[ref16] Landrum G. RDKit: open-source cheminformatics, 2016, http://www.rdkit.org/. 10.5281/zenodo.7415128.

[ref17] Huber F.; Verhoeven S.; Meijer C.; Spreeuw H.; Castilla E. M. V.; Geng C.; van der Hooft J. J.; Rogers S.; Belloum S.; Diblen F.; Spaaks J. H. matchms-processing and similarity evaluation of mass spectrometry data. Journal of Open Source Software 2020, 5 (52), 2411 10.21105/joss.02411.

[ref18] Harris C. R.; Millman K. J.; van der Walt S. J.; Gommers R.; Virtanen P.; Cournapeau D.; Wieser E.; Taylor J.; Berg S.; Smith N. J.; Kern R.; Picus M.; Hoyer S.; van Kerkwijk M. H.; Brett M.; Haldane A.; Del Río J. F.; Wiebe M.; Peterson P.; Gérard-Marchant P.; Sheppard K.; Reddy T.; Weckesser W.; Abbasi H.; Gohlke C.; Oliphant T. E. Array programming with NumPy. Nature 2020, 585 (7825), 357–362. 10.1038/s41586-020-2649-2. 32939066 PMC7759461

[ref19] McKinney W. Data structures for statistical computing in python. In Proceedings of the 9th Python in Science Conference; 2010, Vol. 445, No. (1), , pp 51–56.

[ref20] Virtanen P.; Gommers R.; Oliphant T. E.; Haberland M.; Reddy T.; Cournapeau D.; Burovski E.; Peterson P.; Weckesser W.; Bright J.; van der Walt S. J.; Brett M.; Wilson J.; Millman K. J.; Mayorov N.; Nelson A. R. J.; Jones E.; Kern R.; Larson E.; Carey C. J.; Polat İ; Feng Y.; Moore E. W.; VanderPlas J.; Laxalde D.; Perktold J.; Cimrman R.; Henriksen I.; Quintero E. A.; Harris C. R.; Archibald A. M.; Ribeiro A. H.; Pedregosa F.; van Mulbregt P. SciPy 1.0: fundamental algorithms for scientific computing in Python. Nat. Methods 2020, 17 (3), 261–272. 10.1038/s41592-019-0686-2. 32015543 PMC7056644

[ref21] Mellor C. L.; Marchese Robinson R. L.; Benigni R.; Ebbrell D.; Enoch S. J.; Firman J. W.; Madden J. C.; Pawar G.; Yang C.; Cronin M. T. D. Molecular fingerprint-derived similarity measures for toxicological read-across: Recommendations for optimal use. Regul. Toxicol. Pharmacol. 2019, 101, 121–134. 10.1016/j.yrtph.2018.11.002. 30468762

[ref22] Kind T.; Tsugawa H.; Cajka T.; Ma Y.; Lai Z.; Mehta S. S.; Wohlgemuth G.; Barupal D. K.; Showalter M. R.; Arita M.; Fiehn O. Identification of small molecules using accurate mass MS/MS search. Mass Spectrom. Rev. 2018, 37 (4), 513–532. 10.1002/mas.21535. 28436590 PMC8106966

