# Supplementary material for: Weighting Low-Intensity MS/MS Ions and m/z Frequency for Spectral Library Annotation
Source: J Am Soc Mass Spectrom. 2024 Jan 25;35(2):266–74. doi: 10.1021/jasms.3c00353 (PMC10854760; doi:10.1021/jasms.3c00353)
Supplement: Supplementary file 1 — js3c00353_si_001.pdf [file js3c00353_si_001.pdf]

Supplementary File: Weighting low-intensity MS/MS ions and m/z frequency for spectral library annotation

Chloe Engler Hart[1], Tobias Kind[1], Pieter C. Dorrestein[2], David Healey[1,†*] and Daniel Domingo-Fernández[1,†*]

1. Enveda Biosciences, 5700 Flatiron Parkway, Boulder, CO, 80301, USA

2. Collaborative Mass Spectrometry Innovation Center, Skaggs School of Pharmacy and Pharmaceutical Sciences, University of California San Diego, La Jolla, CA, 92093, USA

† These authors contributed equally to this work.

***Corresponding Authors**: David Healey (david.healey@envedabio.com) and Daniel Domingo-Fernández (dani@envedabio.com), Enveda Biosciences, Boulder, CO, 80301, USA.

Supplementary Figures

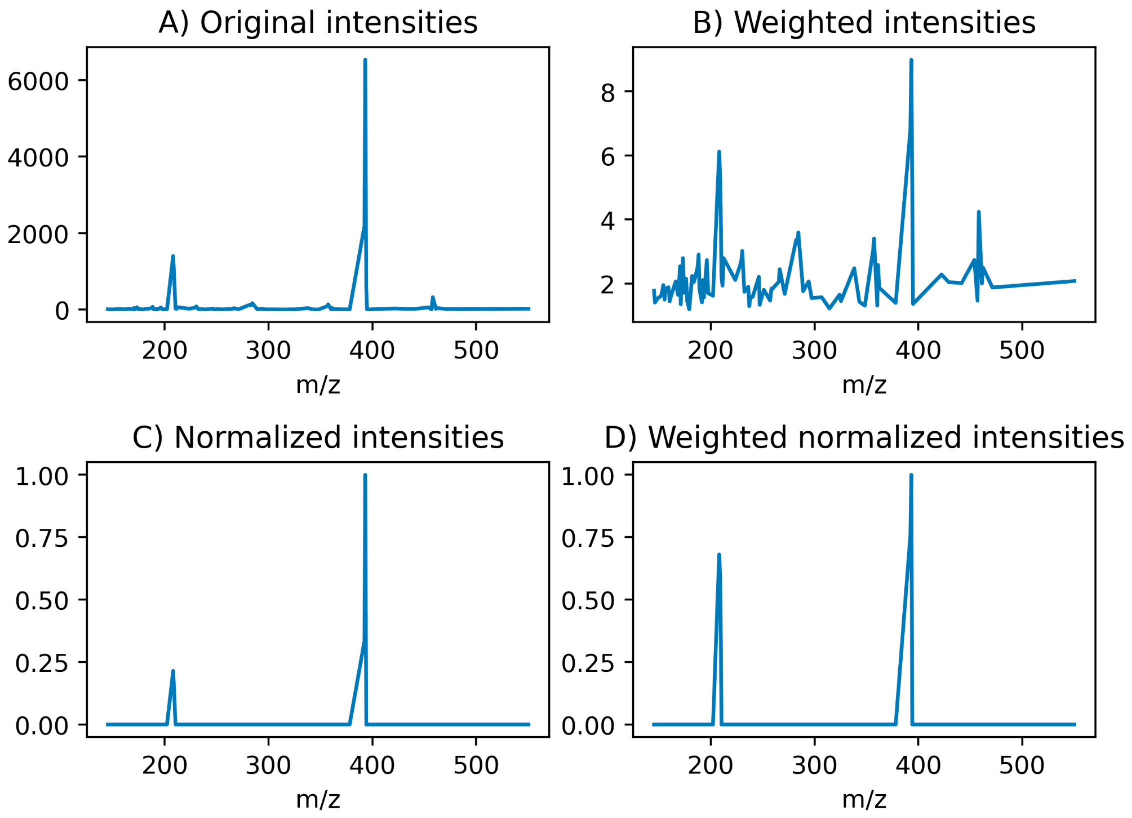

Supplementary Figure 1. Comparing Intensity weighting functions with and without normalization. A) Original intensities for a spectrum. B) Weighted intensities without normalization. C) Normalized intensities for spectrum. D) Weighted intensities after normalization. Normalizing the peaks first allows us to weight the intensities relative to the highest peak.

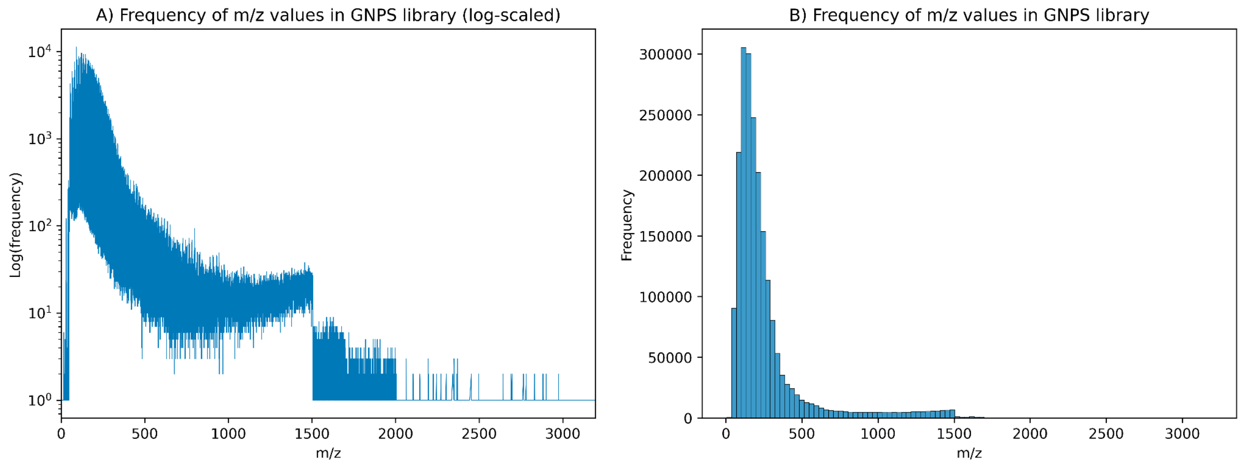

Supplementary Figure 2. Frequency of m/z values in the GNPS library (log scaled (A) and not-scaled (B)). M/z are rounded to 1 decimal place.

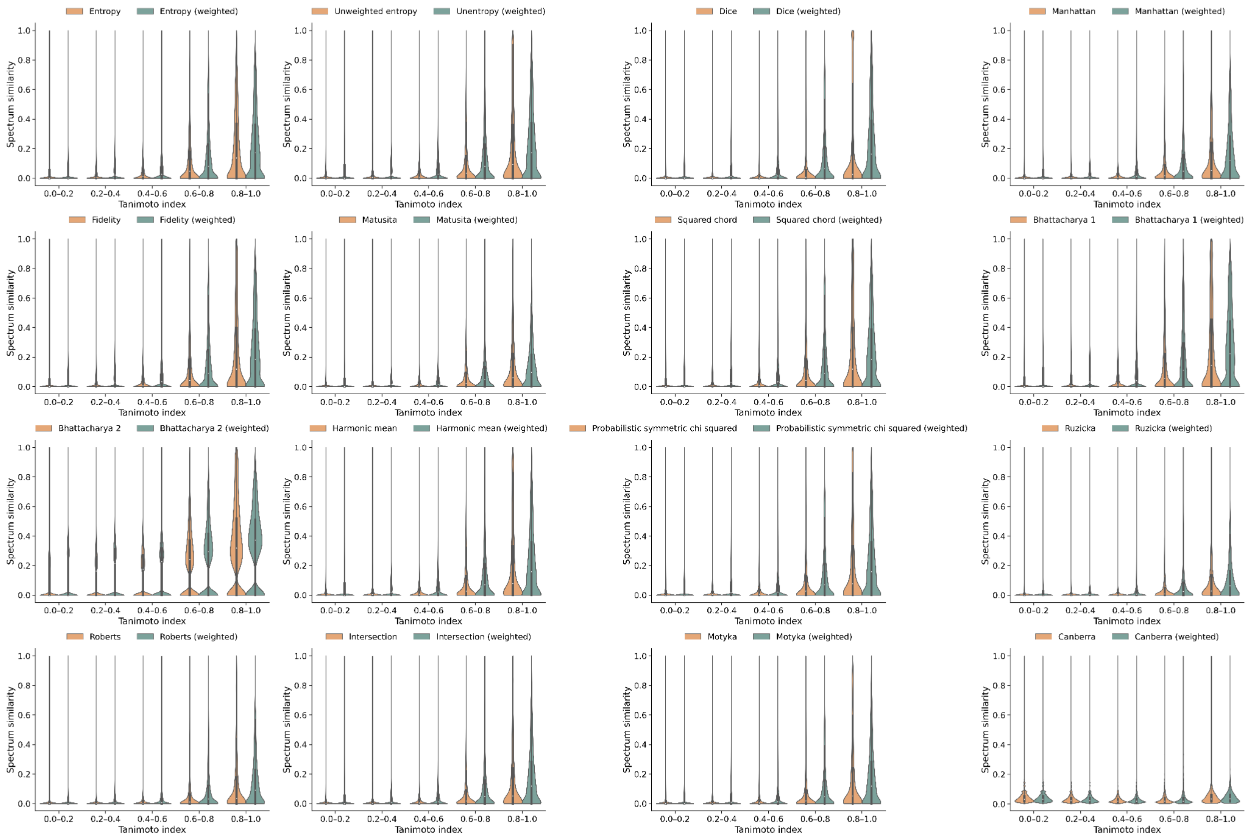

Supplementary Figure 3. Spectral similarities on the 10 million pairs of spectra from GNPS using a subset of the 16 best-performing similarity metrics. The X axis is binned in relation to different ranges of structural similarities measured using the Tanimoto coefficient. The Tanimoto coefficient ranges from 0 to 1, where zero indicates that the pair of molecules has low structural similarity and 1 indicates high structural similarity. Other

metrics such as Euclidean, Chebyshev, Squared Euclidean, Pearson correlation, Lorentzian, Pensore shape, Clark, Hellinger, and Symmetric chi squared are not shown as the spectral similarities did not significantly vary based on the structural similarity, and thus, are not useful for identifying structurally-related compounds. Their True Positive Rate (TPR) and False Positive Rate with the optimized F-1 score are found in **Supplementary Table 4.**

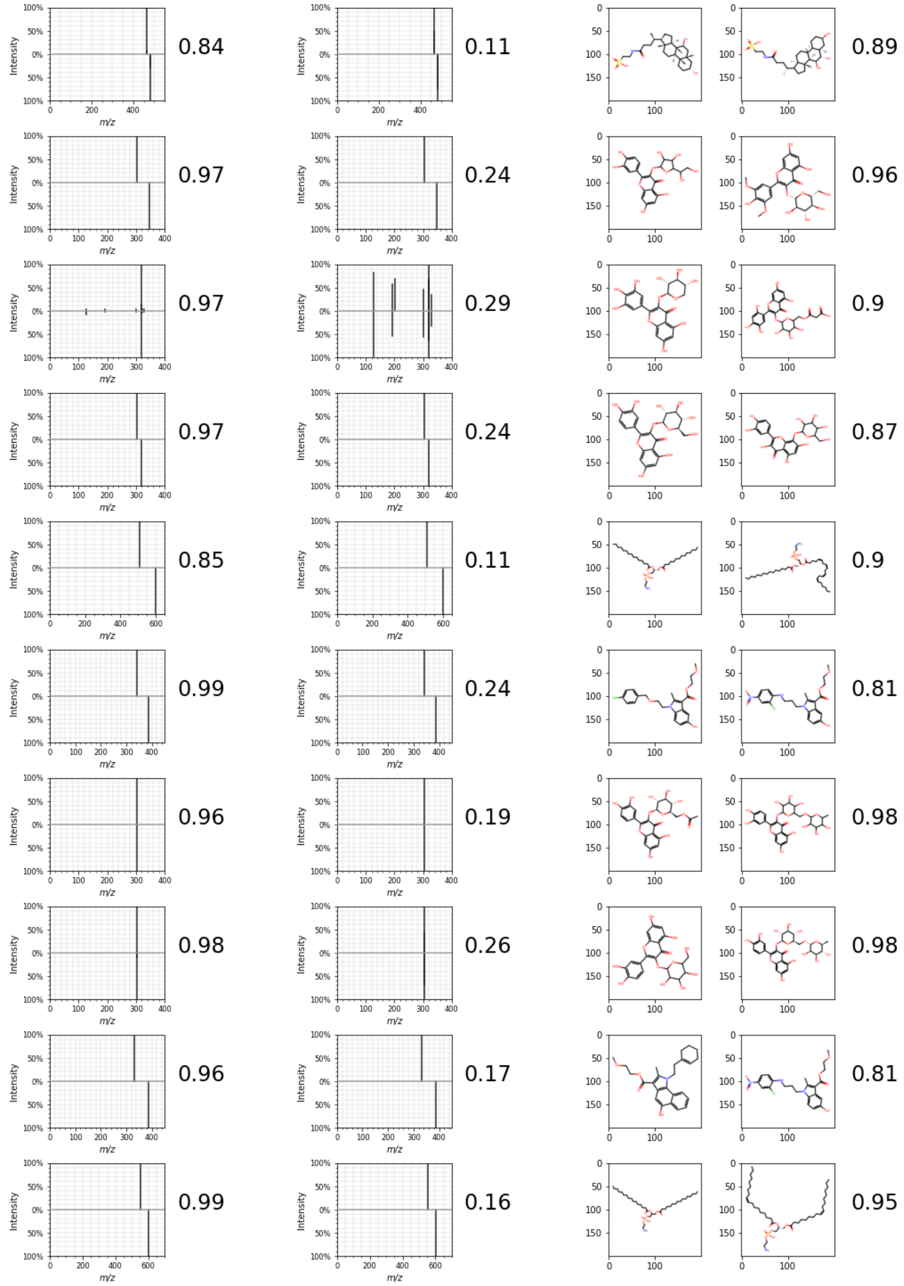

Supplementary Figure 4. Top 10 pairs of spectra where the weights worse the results compared with the original cosine similarity scores. The first column shows the aligned spectra without applying weights on modified cosine similarity and its respective score. Second column shows the aligned spectra after applying weights on modified cosine similarity and its respective score. The third column shows the pairs of compounds whose spectra are analyzed and their Tanimoto coefficient.

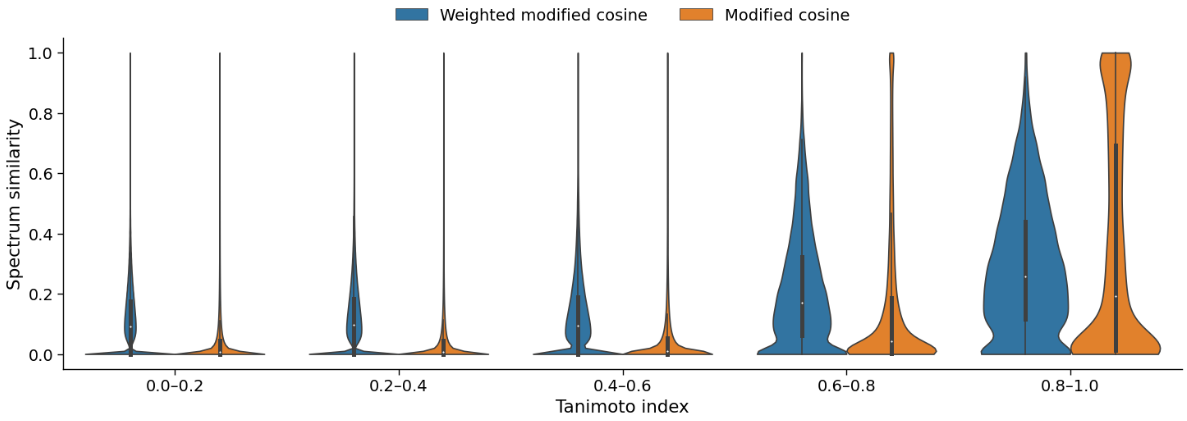

Supplementary Figure 5. Spectral similarities on the 10 million pairs of spectra from GNPS after binarizing all the intensities to 1. The X axis is binned in relation to different ranges of structural similarities measured using the Tanimoto coefficient. The Tanimoto coefficient ranges from 0 to 1, where zero indicates that the pair of molecules has low structural similarity and 1 indicates high structural similarity.

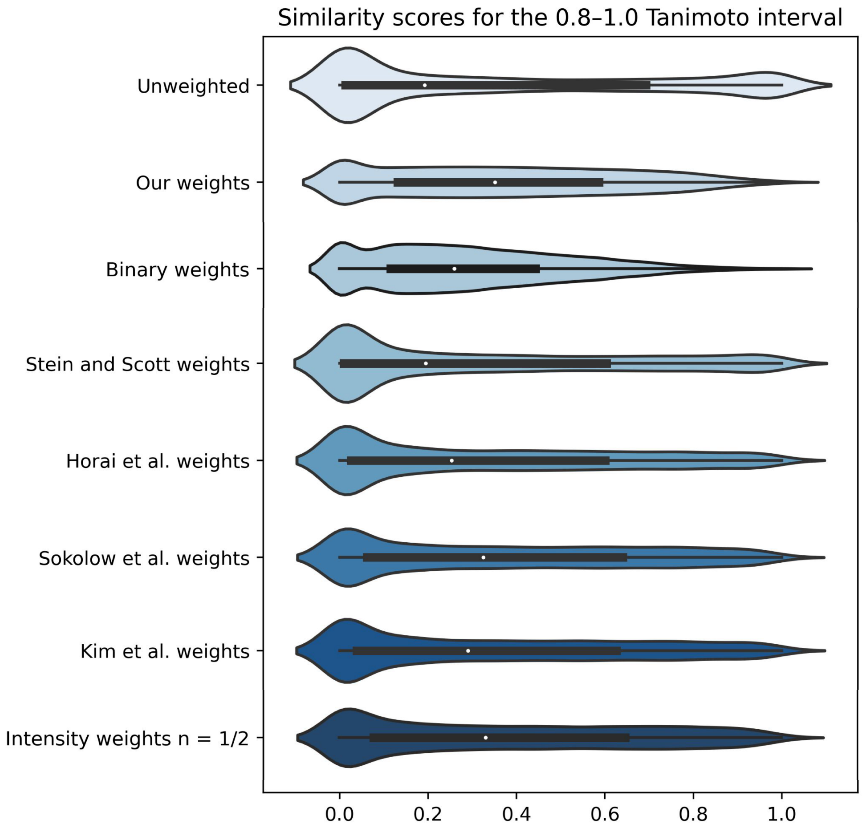

Supplementary Figure 6. Spectral similarities for the high-structurally similar pairs on the 10 million pairs of spectra from GNPS (Tanimoto coefficient > 0.8) using different sets of weights previously reported in the literature. Supplementary Table 3 shows the KL divergence between these distributions and the original distribution of Tanimoto coefficient. Our proposed weights yield the most similar spectral similarities compared to the Tanimoto coefficients.

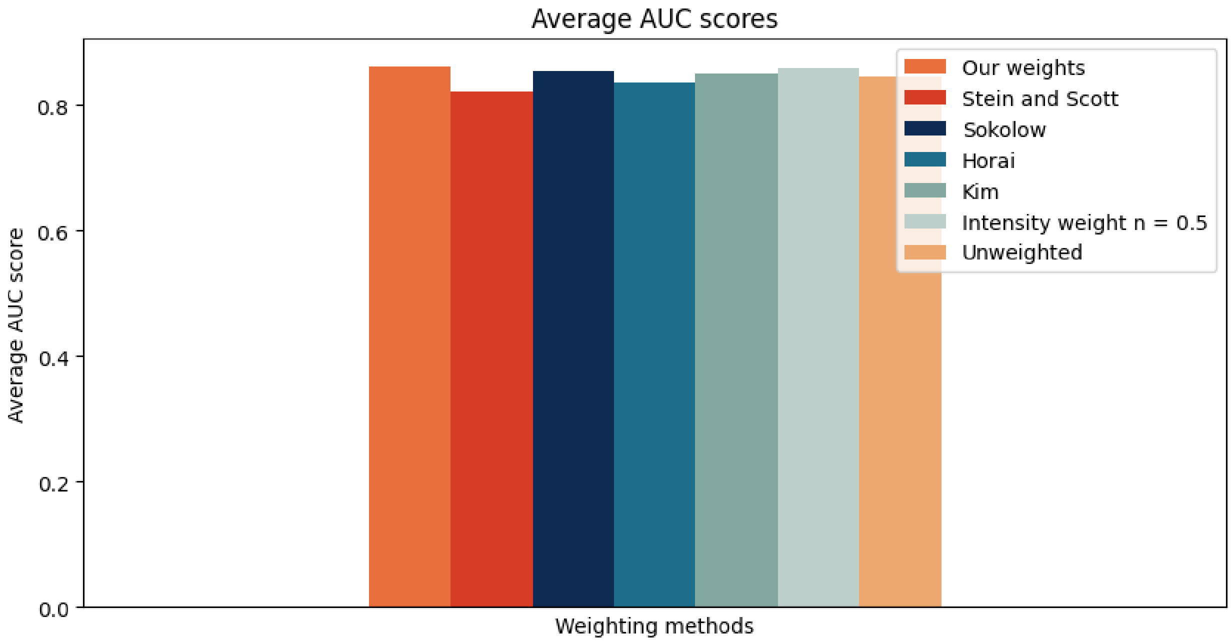

Supplementary Figure 7. Average AUC-ROC scores achieved on the library search task.

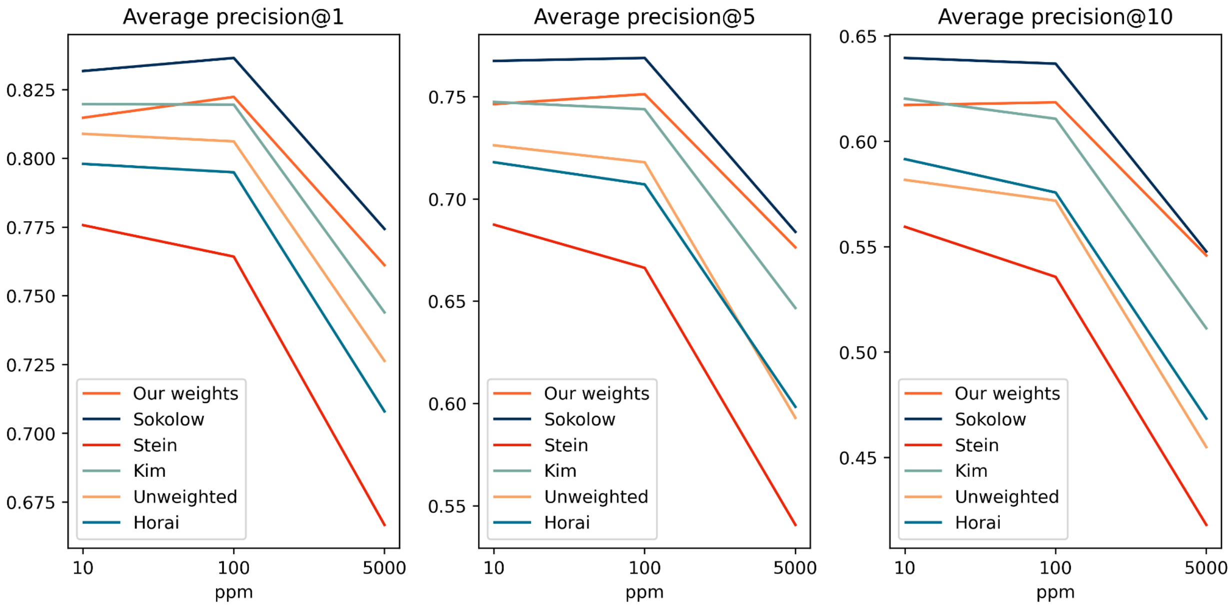

Supplementary Figure 8. Average precision@K across different ppms on the library search task.

Supplementary Tables

Metrics benchmarked in the spectral alignment task		
Spectral entropy	Roberts distance	Penrose shape distance
Unweighted entropy	Motyka distance	Clark distance
Euclidean distance	Canberra distance	Hellinger distance
Manhattan distance	Baroni-Urbani-Buser distance	Whittaker index of association distance
Chebyshev distance	Penrose size distance	Symmetric $\chi 2$ distance
Squared Euclidean distance	Mean character distance	Pearson/Spearman Correlation Coefficient
Fidelity distance	Lorentzian distance	Improved Similarity
Matusita distance	Cosine distance	Absolute Value Distance
Squared-chord distance	Reverse dot product distance	Dot product distance
Bhattacharya 1 distance	Spectral Contrast Angle	Divergence distance
Bhattacharya 2 distance	Wave Hedges distance	Avg (L1, L∞) distance
Harmonic mean distance	Jaccard distance	Vicis-Symmetric $\chi 2$ 3 distance
Probabilistic symmetric $\chi 2$ distance	Dice distance	MSforID distance version 1
Ruzicka distance	Inner product distance	MSforID distance
Weighted dot product distance	Modified cosine similarity	

Supplementary Table 1. Metrics used in spectral alignment task.

Filtering step	Description	Dataset
Quality	Library quality <= 3 (highest quality)	GNPS
Intensity filter	Peaks with at least 1% of the relative intensity	GNPS/NIST
Number of peaks	Minimum 6 (before intensity filter), maximum 200 (after intensity filter)	GNPS/NIST
Adducts	Only [M+H]+ were considered	GNPS/NIST
Precursor charge	Only MS/MS spectra with precursor charge 1 were considered (since only [M+H]+ adducts were considered	GNPS/NIST
Centroid	All intensities > 0	GNPS/NIST
Mode	Positive	GNPS/NIST
Metadata	Structural information required	GNPS / NIST
SMILES/InChIKey	Spectra had a SMILES string or InChiKey	GNPS/NIST
Precursor m/z	Precursor m/z > 0	GNPS/NIST
Spectral similarities	0.1 m/z fragment mass tolerance	GNPS
Precursor m/z ppm	Only spectra with abs(ppm) < 100 were kept (ppm was calculated for spectra using theoretical precursor m/z)	GNPS spectra used in library search
Library	Remove any spectra from the ‘GNPS-NIST14-MATCHES’	GNPS spectra used in library search

Supplementary Table 2. Spectra quality control and filtering.

Weighting Method	KL Divergence
Without weights	3.0563
**Our weights**	**1.1198**
Our intensity weights without *m/z* weights	1.1616
Intensity weight of n = ½ without *m/z* weights	1.5067
Stein and Scott weights	2.5410
Horai *et al.* weights	2.0176
Sokolow *et al.* weights	1.5581
Kim *et al.* weights	1.8332
Binary intensities	1.1847

Supplementary Table 3. KL divergence values for each of the weighting schemes. Supplementary Figure 8 shows the underlying distributions.

-	Unweighted		Weighted	
**Metrics**	**TPR**	**FPR**	**TPR**	**FPR**
Spectral entropy	11.26	0.20	11.34	0.22
Bhattacharya 1	3.05	0.02	8.68	0.14
Dice	9.581	0.21	11.31	0.22
Manhattan	12.30	0.25	11.29	0.24
Fidelity	10.92	0.19	10.39	0.19
Matusita	10.91	0.19	10.37	0.19
Squared chord	10.92	0.19	10.39	0.19
Harmonic mean	11.67	0.22	10.00	0.18
Probabilistic symmetric chi squared	11.67	0.22	10.00	0.18
Ruzicka	11.97	0.24	11.30	0.25
Roberts	12.25	0.26	10.58	0.22
Intersection	12.30	0.25	11.29	0.24
Motyka	12.30	0.25	11.29	0.24
Canberra	1.42	0.125	2.21	0.31
Chebyshev	3.82	0.23	5.96	0.66
Squared euclidean	5.80	0.18	10.46	0.99
Pearson correlation	8.99	0.23	8.74	0.2
Lorentzian	10.28	0.19	11.68	0.25
Penrose shape	4.90	0.11	6.51	0.31
Clark	6.87	0.13	6.45	0.1
Hellinger	4.12	0.08	3.45	0.19
Whittaker	3.21	0.07	2.56	0.15
Symmetric chi squared	8.08	2.45	9.83	2.74

Supplementary Table 4. True positive and false positive rates using the optimal spectral similarity cut-offs based on F1-score for each metric.
